# Specific White Matter Tracts and Diffusion Properties Predict Conversion From Mild Cognitive Impairment to Alzheimer’s Disease

**DOI:** 10.3389/fnagi.2021.711579

**Published:** 2021-07-23

**Authors:** David B. Stone, Sephira G. Ryman, Alexandra P. Hartman, Christopher J. Wertz, Andrei A. Vakhtin, Alzheimer’s Disease Neuroimaging Initiative

**Affiliations:** AbbVie, Alzheimer’s Association; Alzheimer’s Drug Discovery Foundation; Araclon Biotech; BioClinica, Inc.; Biogen; Bristol-Myers Squibb Company; CereSpir, Inc.; Cogstate; Eisai Inc.; Elan Pharmaceuticals, Inc.; Eli Lilly and Company; EuroImmun; F. Hoffmann-La Roche Ltd., and its affiliated company Genentech, Inc.; Fujirebio; GE Healthcare; IXICO Ltd.; Janssen Alzheimer Immunotherapy Research & Development, LLC.; Johnson & Johnson Pharmaceutical Research & Development LLC.; Lumosity; Lundbeck; Merck & Co., Inc.; Meso Scale Diagnostics, LLC.; NeuroRx Research; Neurotrack Technologies; Novartis Pharmaceuticals Corporation; Pfizer Inc.; Piramal Imaging; Servier; Takeda Pharmaceutical Company; and Transition Therapeutics.; The Mind Research Network, Lovelace Biomedical Research Institute, Albuquerque, NM, United States

**Keywords:** Alzheimer’s disease, diffusion tensor imaging, support vector machine, mild cognitive impairment, automated fiber quantification, tractography, conversion, biomarker

## Abstract

Identifying biomarkers that can assess the risk of developing Alzheimer’s Disease (AD) remains a significant challenge. In this study, we investigated the integrity levels of brain white matter in 34 patients with mild cognitive impairment (MCI) who later converted to AD and 53 stable MCI patients. We used diffusion tensor imaging (DTI) and automated fiber quantification to obtain the diffusion properties of 20 major white matter tracts. To identify which tracts and diffusion measures are most relevant to AD conversion, we used support vector machines (SVMs) to classify the AD conversion and non-conversion MCI patients based on the diffusion properties of each tract individually. We found that diffusivity measures from seven white matter tracts were predictive of AD conversion with axial diffusivity being the most predictive diffusion measure. Additional analyses revealed that white matter changes in the central and parahippocampal terminal regions of the right cingulate hippocampal bundle, central regions of the right inferior frontal occipital fasciculus, and posterior and anterior regions of the left inferior longitudinal fasciculus were the best predictors of conversion from MCI to AD. An SVM based on these white matter tract regions achieved an accuracy of 0.75. These findings provide additional potential biomarkers of AD risk in MCI patients.

## Introduction

It is estimated that dementia affects 50 million people world-wide, and it is projected that the number could rise to 152 million by 2050 [Alzheimer’s Disease International (ADI), 2018]. Alzheimer’s disease (AD) accounts for approximately two-thirds of all cases of dementia, making it a global health crisis. As the number of cases of AD rise, there is increasing urgency in early detection and intervention, as well as a need to identify those individuals at greatest risk of developing the disease. AD is typically preceded by a prodromal stage of cognitive decline clinically defined as mild cognitive impairment (MCI); however, it is estimated that only 32–38% of elderly patients exhibiting MCI will develop AD (Mitchell and Shiri-Feshki, 2009; Ward et al., 2013). Identifying those MCI patients at risk of conversion to AD may permit clinicians to plan interventions and courses of treatment. However, identifying specific cognitive, behavioral, and neurodegenerative biomarkers that predict AD conversion remains a significant challenge.

The brain changes that characterize AD may appear years before symptoms emerge (Braak et al., 2011). These changes include amyloid-beta deposition and the buildup of tau protein, as well as early neurodegenerative changes including decreased hippocampal volume, enlarged ventricles, and widespread gray matter (GM) atrophy in prefrontal and temporal cortex (Jack et al., 2010). Consequently, several potential biomarkers of these changes have been developed to predict AD conversion (Ewers et al., 2012; Nanni et al., 2018; Zhang and Shen, 2012). Additionally, AD results in significant white matter (WM) degeneration, and increasing evidence suggests that WM changes appear early and independently of GM tissue loss, are associated with increased tau protein concentrations, and reflect cognitive decline (Amlien and Fjell, 2014; Brun and Englund, 1986; Gold et al., 2012). Therefore, brain WM changes may also serve as potential biomarkers of AD conversion.

The central aim of the current study is to identify patterns of WM degeneration that predict conversion of MCI to AD. To accomplish this aim, we employ a unique approach that utilizes diffusion tensor imaging (DTI) data from MCI patients who either convert to AD or do not and apply a machine learning classification technique to detect specific patterns of WM changes that predict AD conversion.

Diffusion tensor imaging is a non-invasive tool that measures water diffusion in the brain, and is particularly useful in revealing the organizational structure of WM by identifying the trajectories of large axonal bundles, or tracts, using tractography (Mori et al., 1999). Patterns of water diffusion within these tracts can reveal changes in WM integrity that may be useful in predicting AD conversion. Four properties of WM water diffusivity are commonly employed: fractional anisotropy (FA), mean diffusivity (MD), axial diffusivity (AxD), and radial diffusivity (RD). FA measures the degree of directionality of water diffusion, and is associated with the cellular and axonal boundaries that delineate intact WM fibers. Low FA values can indicate loss of WM integrity. MD measures mean diffusion rate through tissue, and high MD values suggest axonal loss and demyelination. AxD and RD measure water diffusion parallel and perpendicular to axonal bundles, respectively. Low AxD measures are associated with axonal degeneration while high RD levels are associated with tract demyelination (Winklewski et al., 2018). Our goal is to determine (1) the specific WM tracts and (2) specific diffusion properties that are most predictive of AD conversion.

To accomplish this goal, we trained and tested support vector machine (SVM) classifiers to detect differences between MCI converters and non-converters based on the four diffusion properties from 20 major WM tracts. A unique SVM classifier was developed for each tract and diffusion property separately, allowing us to parse the predictive value of each tract and property individually. Using this approach, we identified several specific patterns of WM degeneration which can aid clinicians and diagnosticians in determining those individuals at risk of developing AD.

## Materials and Methods

### Data Selection, Participant Inclusion and Exclusion Criteria, and Demographics

The Alzheimer’s Disease Neuroimaging Initiative (ADNI), launched in 2004, is a longitudinal multi-site study designed to develop clinical, genetic, biochemical, and neuroimaging biomarkers to detect and monitor cognitive impairment and Alzheimer’s disease progression (Mueller et al., 2008). The study recruits elderly individuals and originally assigns them to one of four study groups based on clinical assessments: a cognitively healthy group (CN), a group with significant memory concern (SMC), a mild cognitive impairment group (MCI, early, or late), and an Alzheimer’s Dementia group (AD). Clinical, biochemical, and neuroimaging data are collected from participants at multiple timepoints during the study. To date, the ADNI project has proceeded in four phases: ADNI1, 2004–2009; ADNI-GO, 2009–2011; ADNI2, 2011–2016; and ADNI3, 2016-present. In the current study, diffusion-weighted imaging (DWI), structural magnetic resonance imaging (MRI), and clinical assessments from the ADNI2/GO and ADNI3 phases were utilized. Full details of the ADNI study design and protocols can be found online^1^.

At the time of neuroimaging data acquisition, all participants met the criteria for a diagnosis of MCI as established by the ADNI protocols. These criteria include: (1) subjective report of memory concern by participant, informant, or clinician, (2) an education-adjusted score on delayed paragraph recall from the Wechsler Memory Scale Logical Memory II test, (3) a score between 24 and 30 on the Mini-Mental State Exam (MMSE), and (4) a Clinical Dementia Rating (CDR) of 0.5 with preserved cognitive and functional performance such that a diagnosis of AD could not be made. In the current study, no differentiations were made between early and late MCI participants.

Participants were divided into two groups: an MCI group which converted to AD (C), and an MCI group which did not convert to AD (NC). The NC group maintained a diagnosis of MCI post data acquisition for at least 23.5 months (mean, 53.07 months; S.D., 24.11), while the C group met the criteria for an AD diagnosis within 15 months post data acquisition (mean, 10.91 months; S.D., 2.33). AD diagnosis criteria included (1) an MMSE score between 20–26 (20–24 for the ADNI 3 protocol), (2) a CDR of 0.5 or 1, and (3) a clinically determined prognosis of probable AD as established by the NINCDS/ADRDA criteria (McKhann et al., 1984; Dubois et al., 2007; Jack et al., 2011). Diagnoses were reviewed and agreed upon by a committee of clinicians.

Participants were excluded if DWI processing failed to result in diffusion property values for all white matter tracts evaluated (see section “Image Acquisition and Preprocessing”). Based on these criteria, 53 NC and 34 C participants (87 total) were included in the current study. Table 1 summarizes group demographics. No significant group differences in age [mean, 75.1 years; S.D., 8.0 years; *t*(85) = 1.146; *p* = 0.26] or sex [32 female; X^2^(1, *N* = 87) = 2.25; *p* = 0.11] were detected. There was a significant difference in MMSE scores between groups at the time of image acquisition with NC participants showing a small but highly significant greater average score [NC mean, 28.2; S.D., 1.7; C mean, 26.7; S.D., 1.7; *t*(85) = 4.07, *p* < 0.001]. Participants’ medical histories were reviewed for cardiovascular risk and/or disease, including a history of hypertension, high cholesterol, diabetes, coronary disease, or cardiac events. Eleven C and 14 NC participants had a history of such conditions with no significant group differences detected [X^2^(1, *N* = 87) = 0.36; *p* > 0.05].

**TABLE 1:** Participant demographics.

**GROUP**	**C**	**NC**
N	34	53
AGE	76.3 (7.7)	74.3 (8.1)
SEX	9 Female	23 Female
MMSE SCORE*	26.7 (1.7)	28.2 (1.7)
PATIENTS W/CARDIOVASCULAR RISK/DISEASE	11	14
TOTAL GM VOLUME* (Proportion of total intracranial vol)	0.39 (0.02)	0.40 (0.03)
TOTAL WM VOLUME* (Proportion of total intracranial vol)	0.28 (0.02)	0.29 (0.03)
WMH BURDEN*	13,316 mm^3^	7741 mm^3^
PERC WMH BURDEN* (of total WM volume)	3.09% (3.4%)	1.81% (1.7%)

*Indicates measures with significant group differences. Values in parentheses represent standard deviations.

Additionally, several measures of structural brain integrity at time of data acquisition were assessed, including total GM and WM volumes (as proportions of estimated total intracranial volume, in mm^3^) and total volumes of white matter hyperintensity burdens (WMH, as total volume in mm^3^ per subject and as subject-specific percentages of total WM volumes), increases of which have been shown to be risk factors for AD and WM tract atrophy (Taylor et al., 2017). There were significant group differences in total GM and WM volumes [*t*(85) = 2.95, *p* = 0.004; *t*(85) = 2.24, *p* = 0.028, respectively] and marginally significant differences in total volume of WMH burden [*p*(85) = 2.36, *p* = 0.045] and percentage of WMH of total WM volume [*p*(85) = 2.34, *p* = 0.047]. Details regarding the estimations of GM, WM, and WMH can be found in the Supplementary Material.

### Image Acquisition and Preprocessing

Because ADNI is a multi-site study and data were collected across separate study phases, differences existed in image acquisition parameters. All ADNI2 data were acquired on 3-tesla General Electric Medical Systems MR scanners. T1-weighted anatomical scans were acquired using an accelerated fast spoiled gradient echo sequence with inversion recovery (IR-FSPGR) or a magnetization prepared rapid gradient echo (MPRAGE) scan sequence in a 256 × 256 matrix with 1.2 mm × 1.0 mm × 1.0 mm voxel sizes (TE = 2.8–3.0 ms; TI = 400 ms; TR = approx. 7 ms). Axial diffusion-weighted scans were acquired with a spin echo planar imaging sequence in a 256 × 256 matrix with 1.4 mm × 1.4 mm × 2.7 mm voxel sizes (TE = 60–90 ms; TR = 9.05 s). Forty-one diffusion weighted images (*b* = 1,000 s/mm^2^) and five non-diffusion weighted images (*b* = 0 s/mm^2^) were acquired. ADNI3 data were acquired on 3T GE, Siemens, or Philips scanners. T1-weighted accelerated MPRAGE scan sequences were used to acquire anatomical images using the same parameters as ADNI2 scans on GE and Philips systems. Siemens scan sequences were acquired using a 240 × 256 matrix with 1.0 mm^3^ isotropic voxels. Axial diffusion-weighted scans were acquired using a 256 × 256 matrix with 0.9 mm × 0.9 mm × 2.0 mm voxel sizes (GE systems), a 116 × 116 matrix with 2.0 mm^3^ isotropic voxels (Siemens systems), or a 128 × 128 matrix with 2.0 mm^3^ isotropic voxels (Philips systems). Siemens and Philips scans were acquired in 2 mm^3^ isotropic voxels. Between 31 and 127 diffusion-weighted scans were acquired (*b* = 1,000 s/mm^2^), including interleaved non-diffusion weighted (*b* = 0 s/mm^2^) scans with variable echo and relaxation times (depending on scanning site and system). The majority of scans used in the present study were acquired on GE systems (*n* = 70), and no significant differences existed between MCI groups regarding scanner system used [X^2^(2, N = 87) = 0.796, *p* = 0.67].

### Diffusion Weighted Image Processing

For each dataset, diffusion weighted images were registered to the mean non-diffusion weighted (*b* = 0 s/mm^2^) image and corrected for motion and eddy-current artifacts using a 14-parameter deformation algorithm (Rohde et al., 2004). Corrected diffusion images were then aligned to T1-weighted structural images and resampled to 2 mm^3^ isotropic resolution. Diffusion tensors were then fit using the Robust Estimation of Tensors by Outlier Rejection (RESTORE) algorithm (50 iterative steps; Chang et al., 2005). The open-source MrDiffusion software toolbox, part of the Vistasoft software package, was used for DWI preprocessing^2^.

White matter tractography was performed using the Automated Fiber Quantification (AFQ) pipeline (Yeatman et al., 2012). First, whole brain fiber tractography was applied to the tensor fit diffusion weighted data using a deterministic streamline tracking algorithm. Streamlines were seeded at all voxels with a fractional anisotropy (FA) value greater than 0.3 within a WM mask, and tracking terminated when the FA value at the next step was below 0.1 or the angle of the next step direction was greater than 30°. Second, streamlines were assigned to a fiber group if they passed through two waypoint ROIs defined anatomically in Montreal Neurological Institute standard space by Wakana et al., 2007, and transformed into individuals’ native space. The resulting fiber groups were then compared to fiber tract probability maps of major white matter tracts (Hua et al., 2008) transformed into native space, and fibers passing through low probability areas were discarded. Finally, WM tracts were cleaned by excluding fibers greater than four standard deviations above mean fiber length or those which deviated more than five standard deviations from the WM tract core trajectory. This procedure resulted in 20 major WM tracts delineated for each dataset. Each of the 20 WM fiber tracts were segmented into 100 equidistant slices (nodes) and four diffusion properties were calculated at each node: FA, MD, RD, and AxD. The values of these properties at each node were calculated using spline interpolation and summarized as a weighted sum of the values of each fiber contributing to the tract at that node. Weights are based on the distance of each fiber from the center of the tract. Note that, given the variable lengths of the 20 WM tracts, distances between nodes will differ from tract to tract (i.e., in shorter tracts, nodes will be closer together than in longer tracts).

### Classification Based on Diffusion Properties From Single White Matter Tracts

**FIGURE 1:**
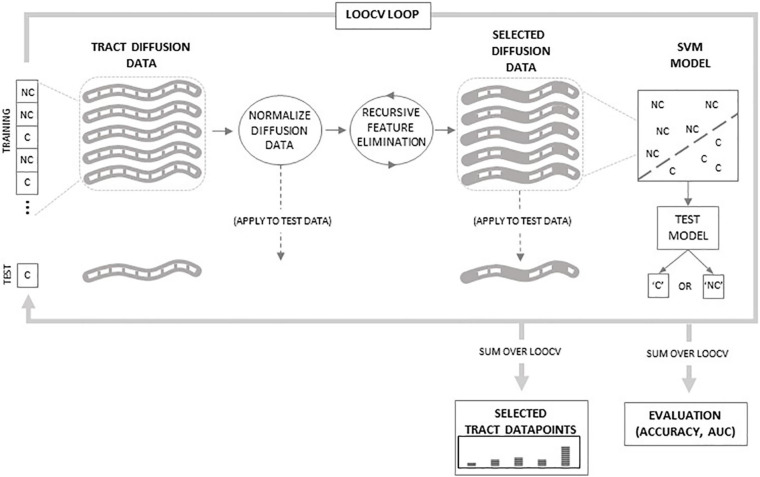
Schematic of classification procedure. Within the leave-one-out cross-validation (LOOCV) loop, tract diffusion data from N-1 subjects are normalized and then recursive feature elimination is applied to select specific datapoints used to train the support vector machine (SVM) model. The model is then tested on the tract data from the remaining subject. The LOOCV loops over all N subjects. Outside of the loop, classifications are summed to evaluate SVM accuracy and area under the curve (AUC) values. Additionally, selected datapoints are summed to determine which regions along the tract are selected by the RFE procedure.

The predictability of each diffusion property of each white matter tract was assessed separately by training and testing a unique support vector machine (SVM) classifier for each tract and diffusion property. Additional SVM classifiers were also trained and tested based on the combined diffusion properties for each tract. All SVMs used for classification were non-linear and used a radial basis function kernel. We chose the non-linear radial basis function kernel SVM because it allows potentially non-linearly separable features to become separable in a higher-dimensional space and can perform better than traditional linear SVMs (Jin and Wang, 2012). Each SVM was trained and tested using the leave-one-out cross-validation (LOOCV) method. LOOCV is a method where an SVM is trained using all but one dataset. The SVM is then tested on the remaining dataset and either correctly or incorrectly classifies that dataset. LOOCV is applied iteratively, leaving one dataset out of training at a time, until all datasets are tested. Figure 1 outlines the SVM training and testing procedure. Within each iteration of the LOOCV loop, the training data are comprised of the N-1 tract diffusion datasets (where N equals the number of subjects) defined by the tract diffusion value at each node along the tract. To ensure proper SVM performance, the training data were normalized by subtracting the mean of each tract node (averaged across training datasets) and dividing by the tract node standard deviation. Normalization was then applied to the testing dataset by subtracting the training dataset means and dividing by the training dataset standard deviations. Recursive feature elimination (RFE) was then applied to the training data to reduce dimensionality and optimize classifier performance. RFE is a common feature selection procedure which reduces the number of features (i.e., tract nodes) used in SVM classification by an iterative process where redundant and uninformative features are eliminated until the subset of features with the highest discriminability between classes is determined (Guyon et al., 2002). In our case, we employed an algorithm developed by Yan and Zhang, 2015, which combines RFE with correlation bias reduction. RFE was applied iteratively to the training data until a subset of the 10 most discriminative tract nodes were derived. These selected tract diffusion features (nodes) were then used to build the SVM classifier model. This SVM model was applied to the testing data based on these selected features and classified the data as either C or NC.

### Evaluation of WM Tract Diffusion Properties

The predictability of the diffusion properties of each WM tract was evaluated based on two metrics of SVM classifier performance: accuracy and the area under the Receiver Operating Characteristic (ROC) curve, or AUC. A true positive occurred when the SVM classifier correctly classified a C dataset and a true negative occurred when the classifier correctly classified an NC dataset. Accuracy was defined as the proportion of correctly classified datasets (i.e., the sum of true positives and true negatives) summed over all testing datasets (LOOCV loops). AUC is a measure that quantifies the trade-off between true and false positives (i.e., correctly classified C datasets vs. incorrectly classified NC datasets) where values greater than 0.5 reflect a higher ratio of true positives.

Once classifier accuracies and AUCs were obtained for every diffusion property from every WM tract, selection criteria were applied to further identify those tracts and properties that possessed predictive value. A random classifier performing at chance will obtain an accuracy greater than 0.59 with a probability 0.05 or less (based on the number of datasets in the current study). Additionally, a classifier that obtains an AUC at or near 0.5 is also performing at chance. Therefore, only tract and diffusion properties that obtained accuracies greater than expected by chance (at *p* < 0.05) and which also possessed AUC values greater than 0.6 were considered predictive of AD conversion.

To test the statistical significance of the accuracies obtained from these predictive tracts and diffusion properties, permutation tests were performed. In these tests, the accuracy of each classifier was compared to a distribution of accuracies obtained from 1,000 classifiers that used the same tract diffusion data, but in which group assignment (C or NC) was randomly permuted in the same proportion as the original data. For each permuted classifier, normalization and RFE procedures were performed to ensure consistency. A one-sample *t*-test was used to compare the accuracy obtained from the SVM classifier to the distribution of accuracies obtained from the 1,000 permutations. Values less than *p* = 0.05 were considered significant.

Because we applied the RFE optimization procedure to select the 10 most discriminative tract nodes in each iteration of the LOOCV loop, we were able to obtain a histogram of the most selected nodes from each WM tract based on each diffusion property. This allowed us to identify the regions in each WM tract that were most predictive of AD conversion. Once these nodes were identified, standard statistical tests were performed to determine if significant C vs. NC group differences existed in the diffusion values at these nodes. Independent samples *t*-tests were performed at each node where the false discovery rate was controlled to adjust for multiple comparisons (*p* < 0.05).

Tract nodes selected by the most accurate classifiers which also showed significant group differences were considered WM tract regions most predictive of AD conversion. To further confirm and quantify the predictability of these regions, a final SVM classifier was trained and tested. This classifier followed the same classification procedure outlined above; however, it utilized only the data from the selected and significant nodes. The accuracy and AUC of this classifier quantified the final predictive value of the specific tracts, diffusion properties, and tract regions implicated in AD conversion.

## Results

Tables 2, 3 display the accuracies and AUCs obtained from all tract diffusion property classifications, respectively. Of the 80 single tract and diffusion property classification tests performed, 10 were found to possess predictive value (i.e., obtained an accuracy > 0.59 and an AUC > 0.6). Permutation tests confirmed that these 10 tract and diffusion properties were significantly accurate in discriminating between C and NC (*p* < 0.0001, all tests). It should be noted that radial diffusivity of the right arcuate fasciculus tract also possessed predictive value, obtaining an accuracy of 0.60; however, the AUC was 0.35. This suggests that, while poor at classifying AD converters, radial diffusivity from this tract classified non-converters well.

**TABLE 2:** SVM classifier accuracies from single tract classification.

**ACCURACY**					
**TRACT**	**FA**	**MD**	**RD**	**AxD**	**ALL**
Left thalamic radiation	0.63	0.46	0.49	0.55	0.52
Right thalamic radiation	0.61	0.52	0.53	0.45	0.54
Left corticospinal	0.61	0.49	0.55	0.67*	0.59
Right corticospinal	0.55	0.54	0.48	0.61*	0.52
Left cingulum cingulate	0.58	0.51	0.43	0.47	0.57
Right cingulum cingulate	0.56	0.54	0.49	0.60	0.51
Left cingulum hippocampus	0.59	0.52	0.48	0.52	0.59
Right cingulum hippocampus	0.55	0.68**	0.54	0.67**	0.59
Callosum forceps major	0.59	0.55	0.56	0.61	0.51
Callosum forceps minor	0.61	0.59	0.55	0.62	0.59
Left IFOF	0.59	0.55	0.56	0.54	0.59
Right IFOF	0.59	0.58	0.55	0.72**	0.59
Left ILF	0.59	0.60**	0.62**	0.63**	0.59
Right ILF	0.52	0.53	0.56	0.62	0.51
Left SLF	0.61	0.54	0.60	0.58	0.61
Right SLF	0.56	0.56	0.59	0.68*	0.54
Left uncinate	0.53	0.67*	0.58	0.56	0.62
Right uncinate	0.61	0.58	0.59	0.56	0.51
Left arcuate	0.62	0.52	0.55	0.59	0.54
Right arcuate	0.61	0.55	0.60**^‡^**	0.55	0.54

*Indicates accuracy with predictive value of AD conversion.

**Indicates tract diffusion property where statistically significant group differences at selected nodes were detected.

^‡^*Indicates accuracy with predictive value of non-conversion to AD.*

**TABLE 3:** SVM classifier areas under curves (AUCs) from single tract classification.

**AUC**					
**TRACT**	**FA**	**MD**	**RD**	**AxD**	**ALL**
Left thalamic radiation	0.58	0.38	0.55	0.48	0.44
Right thalamic radiation	0.55	0.36	0.25	0.43	0.40
Left corticospinal	0.47	0.37	0.39	0.71*	0.52
Right corticospinal	0.41	0.39	0.41	0.61*	0.44
Left cingulum cingulate	0.53	0.35	0.40	0.39	0.52
Right cingulum cingulate	0.43	0.44	0.56	0.41	0.51
Left cingulum hippocampus	0.49	0.49	0.43	0.49	0.55
Right cingulum hippocampus	0.33	0.72**	0.61	0.68**	0.61
Callosum forceps major	0.45	0.53	0.51	0.53	0.41
Callosum forceps minor	0.48	0.47	0.24	0.58	0.45
Left IFOF	0.44	0.56	0.51	0.54	0.56
Right IFOF	0.51	0.53	0.56	0.67**	0.50
Left ILF	0.44	0.61**	0.67**	0.65**	0.59
Right ILF	0.40	0.53	0.59	0.53	0.42
Left SLF	0.41	0.45	0.54	0.50	0.57
Right SLF	0.48	0.64	0.59	0.72*	0.46
Left uncinate	0.23	0.61*	0.51	0.49	0.59
Right uncinate	0.44	0.45	0.64	0.57	0.49
Left arcuate	0.53	0.40	0.32	0.49	0.43
Right arcuate	0.42	0.41	0.35**^‡^**	0.43	0.24

*Indicates accuracy with predictive value of AD conversion.

**Indicates tract diffusion property where statistically significant group differences at selected nodes were detected.

^‡^*Indicates accuracy with predictive value of non-conversion to AD.*

The WM tracts where at least one diffusion property had predictive value include the left and right corticospinal tracts, the right cingulum hippocampal bundle (right CHB), the right inferior frontal occipital fasciculus (right IFOF), the left inferior longitudinal fasciculus (left ILF), the right superior longitudinal fasciculus (right SLF), and the left uncinate fasciculus.

Of the four diffusion properties, AxD accounted for 60% (6 out of 10) of the diffusion measures from tracts with predictive value, followed by MD (3 out of 10) and RD (1 out of 10). FA from all 20 tracts evaluated failed to predict AD conversion greater than chance, based on accuracy and AUC. Interestingly, single-tract SVM classifiers based on all diffusion properties were less predictive than classifiers based on a single diffusion property for each of the 20 tracts evaluated, and none performed better than chance.

As an additional exploration of the results obtained from our classification tests, we decided to evaluate potential sex differences in the accuracies of each tract and diffusion property. Although our sample only included nine C and 23 NC female participants, we found that accuracies were greater for females than males and greater than overall (male and female combined) accuracies on almost every tract and diffusion property classification. Accuracies according to participant sex are presented in Supplementary Table 1.

**FIGURE 2:**
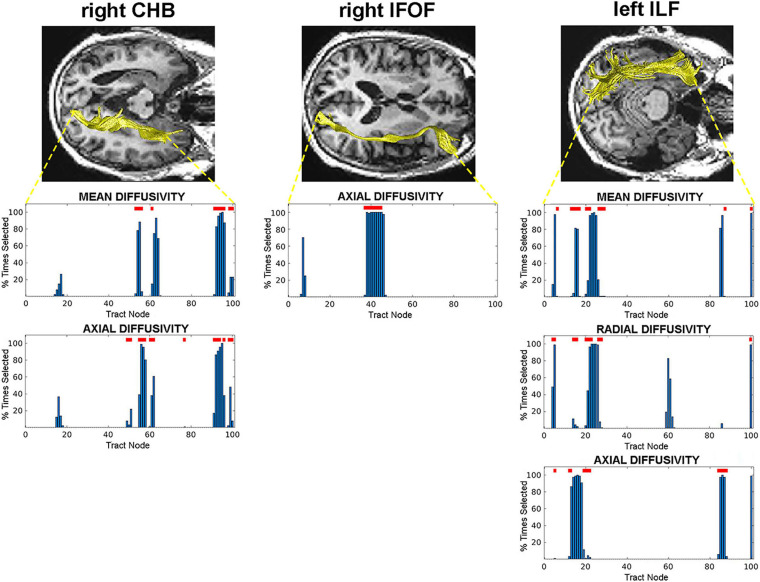
Tract nodes selected by the RFE procedure for tracts with predictive value. Top images display tracts from one participant overlaid on structural images in axial view. Bottom graphs are histograms displaying the percentage of times each tract node was selected by the RFE procedure. Red lines above bars indicate nodes where significant C vs. NC group differences were detected. Left: right CHB tract; Middle: right IFOF tract; Right: left ILF tract.

We identified the specific nodes along each tract that predicted AD conversion for each diffusion property that possessed predictive value. These were the specific nodes selected during SVM classification training by the RFE process. We then evaluated the diffusion values at each of these selected nodes to determine where significant C vs. NC group differences existed. This analysis revealed specific regions (groups of nodes) in three WM tracts that are most predictive of AD conversion. Specifically, we found central and terminal (hippocampal) regions in the right CHB tract; a central region in the right IFOF tract; and posterior and anterior regions in the left ILF tract (Figure 2). These regions were identified across multiple diffusion measures in the right CHB and left ILF tracts.

As a final measure of the predictive value of these selected and significant WM regions, a final SVM classifier was trained and tested using only the diffusion data at these specific nodes. This classifier achieved an accuracy of 0.75 and an AUC of 0.72. The RFE process from this final classifier revealed that nodes from all three WM tracts were selected: node 96 in the right CHB (mean diffusivity), nodes 37–45 in the right IFOF, and node 86 in the left ILF (axial diffusivity).

## Discussion

In the current study, tractography-based SVM classifiers were used to evaluate differences between stable MCI patients and those who converted to AD based on four diffusion properties from 20 major WM tracts. A classifier was developed for each diffusion property from each WM tract independently, so that the predictive value of individual tracts and diffusion properties could be evaluated separately. Results revealed seven WM tracts predictive of AD conversion. Axial diffusivity was the most predictive property of AD conversion, followed by mean and radial diffusivities. Additional analyses revealed specific regions along each of these tracts that predicted conversion. When these regions were tested for significant group differences between AD converters and non-converters, regions from three tracts emerged as the most likely indicators of AD conversion risk: central and terminal regions of the right CHB, central regions of the right IFOF, and posterior and anterior regions of the left ILF WM tracts.

In the current study, we found that the right CHB tract, the right IFOF tract, and the left ILF tracts were the most predictive of AD conversion in MCI patients. These tracts connect to GM structures associated with memory function, and WM compromise in these tracts has been associated with increased risk of AD conversion (Fu et al., 2014; Gold et al., 2012; Mielke et al., 2012). In particular, the CHB and ILF tracts are the major connecting fibers of the parahippocampal gyrus (Lin et al., 2021). Our analysis shows that the parahippocampal terminal regions of both of these tracts are compromised in at-risk MCI patients, consistent with previous reports (Mielke et al., 2012; Marcos Dolado et al., 2019). Interestingly, in a study reported by Solodkin et al., 2013, which performed a DTI analysis restricted to the parahippocampal gyrus, the authors found that anterior regions of the gyrus were compromised in MCI patients who converted to AD, while patients with AD had WM compromise throughout the gyrus, suggesting that this region may be affected early during prodromal stages of the disease. The right IFOF connects frontal cortical regions to occipital, posterior temporal, and parietal cortices (Sarubbo et al., 2011). Loss of WM integrity in the IFOF has been implicated as a risk factor for conversion to AD (Bendlin et al., 2010; Smith et al., 2010; Fu et al., 2014), and AD patients exhibit widespread compromise along the IFOF tract, including anterior, posterior, and central regions which is associated with memory impairment (Nir et al., 2015; Chen et al., 2020; Dou et al., 2020). Our results suggest that compromise to the central region of the IFOF may precede more extensive damage and be an early stage of disease progression.

In our study, axial diffusivity was the diffusion measure which was the greatest predictor of AD conversion. Changes in axial diffusivity have been associated with axonal injury or compromise (Song et al., 2003; Mac Donald et al., 2007; Winklewski et al., 2018), and our findings thus suggest that loss of axonal integrity may be an early indicator of AD risk. Of the few studies that have examined DTI measures as potential predictors of AD conversion, most report changes in fractional anisotropy or mean diffusivity (Fu et al., 2014; Brueggen et al., 2015; Dyrba et al., 2015; Makovac et al., 2018; Marcos Dolado et al., 2019), suggesting that changes in mean diffusivity may predict AD conversion. Given that mean diffusivity is a composite measure that reflects both axial and radial diffusivity, it is possible that the predictive value of mean diffusivity is driven by loss of axonal integrity; however, more research is needed. Nevertheless, our results highlight the merit in examining multiple diffusivity measures when identifying patients at risk of AD conversion.

Although not a central focus of our study, the analysis of sex differences in tract and diffusion property classification revealed that differences between C and NC MCI patients were greater for females than males. This is consistent with reports that men and women differ in incidence, pathology, and progression to AD from MCI (Kim et al., 2015; Mazure and Swendsen, 2016). While an intriguing finding, it should be noted that our low sample size of women in the current study (32 females total with nine AD converters) warrants caution in reaching definitive conclusions. As the ADNI project is an on-going study, it is likely that more female MCI patients (converters and non-converters) will emerge, and a clearer picture of sex differences in AD conversion, specifically WM tract changes, will allow clinicians to develop more sex-targeted interventions and treatments.

Additionally, the MCI patients in our study had several significant differences in measures of whole brain GM and WM volumes, as well as differences in WMH burden with the AD conversion group showing greater total lesion volume compared to non-converters. Higher WMH burden has been associated with dementia, including AD, and is often already present in MCI patients (Debette and Markus, 2010). However, the impact that WMH lesions have on WM tract integrity is less clear. In a recent study by Reginold et al., 2018, the authors specifically examined WMH impact on corticospinal WM tracts in a healthy control population. They concluded that a pattern of Wallerian-type degeneration occurs when lesions directly transect WM fibers, impacting the fiber along its length, while fibers near WMH lesions show a penumbra effect, compromising nearby tract integrity with decreasing effect with distance. Since we did not specifically examine interactions between WMH lesions and tract integrity, the WM tract changes we observed could have resulted from either or both possibilities in affected tracts. The degree to which WMH differences between our groups resulted in the WM tract differences we observed remains an intriguing question. The focus of future research will be to determine how WMHs evolve in AD and how they interact with changes in WM tract integrity.

White matter tissue loss in AD has traditionally been regarded as secondary to GM tissue loss (Roher et al., 2002), a view supported by the amyloid cascade hypothesis (Hardy and Higgins, 1992). However, there is evidence that WM damage in AD occurs independently of GM damage, and the relationship between GM and WM loss during disease progression is likely complex. For example, several studies have reported that WM damage may precede hippocampal atrophy during prodromal stages of AD and may be a better predictor of conversion to AD than GM tissue damage (Agosta et al., 2011; Brickman et al., 2012; Zhuang et al., 2013). There is also emerging evidence that WM loss may result in downstream GM neurodegeneration. In a recent study by Araque Caballero et al., 2018, the authors found that specific WM tracts are affected years before symptoms in early-onset autosomal dominant AD, where increased degeneration in callosal and projection WM tracts was associated with increased GM damage in the projection zones of these tracts. This evidence is supported by the discovery that tau pathology is propagated along connected WM pathways and that tau accumulates in downstream regions connected to affected WM tracts (Jacobs et al., 2018). It is reasonable to suspect that interactions between GM and WM tissue damage evolve during AD progression and that different regions will show different neurodegenerative patterns at different stages during the disease. In future research, we intend to extend our findings by investigating degeneration in regions where predictive WM tracts originate and terminate across disease progression, and a clearer pattern of GM and WM interactions in AD progression may emerge.

There is evidence that WM degeneration, as detected non-invasively through DTI, may contribute to other forms of dementia, particularly vascular dementia (Finsterwalder et al., 2020; Vemuri et al., 2018). An intriguing question is the extent to which the biomarkers predictive of AD conversion investigated here are specific to AD or whether they may reflect risk for other forms of dementia. Emerging research which employs DTI measures to parse vascular and AD dementias may shed light on these questions (Raja et al., 2020).

There are several limitations to the current study that should be noted. First, we acknowledge that the number of AD converter and non-converter participants used in our study is small. Unfortunately, despite the increasing application of DTI in longitudinal studies of AD and dementia, there remains a paucity of such data. Indeed, it is a limitation of many studies investigating AD conversion based on DTI metrics. We anticipate that, as research continues, more DTI data will become available, and the results obtained in the present study can be further tested. Nevertheless, we believe that our approach for detecting potential WM changes predictive of AD conversion, which emphasizes individual tract and diffusion property classification, will facilitate the search for effective biomarkers as more data are collected. A second limitation of the current study is that the differences between MCI groups were examined at only one timepoint for each participant. Subtleties in the dynamics of WM changes as they progress from MCI to AD are likely missed. Planned research, which incorporates longitudinal data, may reveal additional vulnerabilities in patients who convert to AD, as well as factors that confer resilience in those patients who do not. Finally, we note that our choice of SVM classifiers is only one of many potential machine learning approaches. Machine learning has been used extensively in recent years for early AD diagnosis, where its utility in recognizing patterns in complex neuroimaging data has been exploited to effectively classify and discriminate between early AD and MCI patients (for recent reviews, see Rathore et al., 2017; Martí-Juan et al., 2020; Tanveer et al., 2020). Although other approaches exist (e.g., logistic regression, artificial neural networks, linear discriminant analyses, Bayes classifiers), several studies which have directly compared machine learning algorithms in AD classification suggest SVM classifiers perform as well as or better than other approaches and remain one of the most widely-used (Cabral et al., 2015; Zhang and Liu, 2018; Tanveer et al., 2020; Wen et al., 2021). Nevertheless, different approaches could yield additional findings regarding WM tract integrity as a potential predictor of AD conversion.

Ongoing research has revealed multiple potential predictors of risk of AD conversion, and several candidate biomarkers have emerged. These include molecular biomarkers of amyloid beta deposition and early neuronal damage, neuroimaging biomarkers of structural GM neurodegeneration and cerebral atrophy, neuropsychological markers of cognitive decline, as well as combinations of multiple markers (Ewers et al., 2012; Zhang and Shen, 2012; Nanni et al., 2018). Our results complement this earlier work and provide additional indicators of potential risk of developing AD. The need to discover definitive biomarkers for AD prediction and diagnosis is most clearly realized by radiologists, clinicians, and diagnosticians whose patients’ care and management depend on their assessments. DTI is a non-invasive means to detect WM changes and, with increasing evidence that WM degeneration is a central component of AD progression, provides additional measures that can facilitate effective and reliable prognoses. Further, with the development and availability of more sophisticated tools, such as automatic tractography, novel WM predictors can be readily incorporated into the diagnostic profile. Hopefully, our results will help guide clinicians as they make determinations about those patients at greatest risk of developing AD.

## Data Availability Statement

Publicly available datasets were analyzed in this study. This data can be found here: Alzheimer’s Disease Neuroimaging Initiative (ADNI) data repository at adni.loni.usc.edu.

## Ethics Statement

Ethical review and approval was not required for the study on human participants in accordance with the local legislation and institutional requirements. The patients/participants provided their written informed consent to participate in this study. Written informed consent was obtained from the individual(s) for the publication of any potentially identifiable images or data included in this article.

## Author Contributions

DS designed the study, developed original software, analyzed the data, and wrote the manuscript. SR conceptualized the work and reviewed and edited the manuscript. AH and CW curated the data, assisted in data processing, and reviewed and edited the manuscript. AV conceptualized the work, contributed to study design, reviewed and edited the manuscript, and supervised the work. All authors contributed to the article and approved the submitted version.

## Conflict of Interest

The authors declare that the research was conducted in the absence of any commercial or financial relationships that could be construed as a potential conflict of interest.

## Publisher’s Note

All claims expressed in this article are solely those of the authors and do not necessarily represent those of their affiliated organizations, or those of the publisher, the editors and the reviewers. Any product that may be evaluated in this article, or claim that may be made by its manufacturer, is not guaranteed or endorsed by the publisher.

## Footnotes

1 http://adni.loni.usc.edu/methods/documents

2 http://web.stanford.edu/group/vista/cgi-bin/wiki/index.php/MrDiffusion
