# Supplementary material for: Specific White Matter Tracts and Diffusion Properties Predict Conversion From Mild Cognitive Impairment to Alzheimer’s Disease
Source: Front Aging Neurosci. 2021 Jul 23;13:711579. doi: 10.3389/fnagi.2021.711579 (PMC8343075; doi:10.3389/fnagi.2021.711579)
Supplement: Supplementary file 1 [file Data_Sheet_1.docx]

**Supplementary Methods**

*Participant Gray Matter and White Matter Volume Estimations*

For each participant, total intracranial volume (including ventricular volume), total cerebral white matter volume, and total gray matter volume were estimated based on each participant’s T1-weighted structural MRI using the Freesurfer Analysis Suite which is documented and freely available for download online (<http://surfer.nmr.mgh.harvard.edu/>).

Briefly, Freesurfer uses an automated process to segment and parcellate the T1-weighted image volume by utilizing normalized image intensity gradients to optimally define boundaries between WM and GM cortical and subcortical structures after automatic removal of non-brain tissue (Fischl et al., 2002). Total intracranial volume, total cerebral white matter volume, and total gray matter volume are then estimated at 1 mm^3^ resolution based on these defined boundaries.

For our analysis, total gray matter volume and total cerebral white matter volume were divided by the total intracranial volume (all values in mm^3^) for each participant to obtain the values for total GM volume and total WM volume which appear in Table 1.

*Estimation of Participant White Matter Hyperintensity Burden*

White matter hyperintensity (WMH) burden for each participant was estimated using the Lesion Segmentation Toolbox (LST) freely available for download at ([www.statisticalmodelling.de/lst.html](http://www.statisticalmodelling.de/lst.html)). LST estimates WMH volumes based on the T2-weighted fluid-attenuated inversion recovery (FLAIR) MR structural images from each participant by using a lesion prediction algorithm. The algorithm utilizes a logistic regression binary classifier model trained on 53 patients with severe lesion patterns and assigns to each voxel in the FLAIR image a probability value (between 0 and 1) that a WMH lesion exists at that voxel (Schmidt 2017, Chapter 6.1).

For our analysis of total WMH volume, we set a probability value threshold of 0.8 and calculated the number of voxels equal to or exceeding this value. We then converted the voxel counts to volumes at 1 mm^3^ resolution to obtain total estimated WMH burden. Percent of total WMH burden for each participant (summarized in Table 1) was then calculated by dividing the total estimated WMH volume by the total cerebral white matter volume (obtained from the Freesurfer segmentation described above).

*References*

Fischl, B., Salat, D.H., Busa, E., Albert, M., Dieterich, M., Haselgrove, C., van der Kouwe, A., Killiany, R., Kennedy, D., Klaveness, S., Montillo, A., Makris, N., Rosen, B., Dale, A.M., 2002. Whole brain segmentation: automated labeling of neuroanatomical structures in the human brain. Neuron 33, 341-355.

Paul Schmidt., 2017. Bayesian inference for structured additive regression models for

large-scale problems with applications to medical imaging. PhD thesis, Ludwig-

Maximilians-Universität München. http://nbn-resolving.de/urn:nbn:de:bvb:19-203731.
