# Supplementary material for: Specific White Matter Tracts and Diffusion Properties Predict Conversion From Mild Cognitive Impairment to Alzheimer’s Disease
Source: Front Aging Neurosci. 2021 Jul 23;13:711579. doi: 10.3389/fnagi.2021.711579 (PMC8343075; doi:10.3389/fnagi.2021.711579)
Supplement: Supplementary file 2 [file Table_1.docx]

| **ACCURACY MALE PATIENTS ONLY** | | | | | |
| --- | --- | --- | --- | --- | --- |
| **TRACT** | **FA** | **MD** | **RD** | **AxD** | **ALL** |
| **Left Thalamic Radiation** | 0.55 | 0.40 | 0.40 | 0.47 | 0.42 |
| **Right Thalamic Radiation** | 0.60 | 0.44 | 0.44 | 0.38 | 0.51 |
| **Left Corticospinal** | 0.60 | 0.45 | 0.51 | 0.64 | 0.55 |
| **Right Corticospinal** | 0.53 | 0.49 | 0.44 | 0.60 | 0.47 |
| **Left Cingulum Cingulate** | 0.51 | 0.49 | 0.42 | 0.42 | 0.51 |
| **Right Cingulum Cingulate** | 0.55 | 0.53 | 0.44 | 0.55 | 0.49 |
| **Left Cingulum Hippocampus** | 0.53 | 0.45 | 0.38 | 0.45 | 0.51 |
| **Right Cingulum Hippocampus** | 0.49 | 0.64 | 0.47 | 0.65 | 0.53 |
| **Callosum Forceps Major** | 0.58 | 0.55 | 0.55 | 0.58 | 0.47 |
| **Callosum Forceps Minor** | 0.56 | 0.56 | 0.51 | 0.60 | 0.51 |
| **Left IFOF** | 0.55 | 0.53 | 0.51 | 0.51 | 0.56 |
| **Right IFOF** | 0.53 | 0.51 | 0.49 | 0.64 | 0.53 |
| **Left ILF** | 0.53 | 0.55 | 0.60 | 0.60 | **0.62** |
| **Right ILF** | 0.45 | 0.47 | 0.49 | 0.56 | 0.49 |
| **Left SLF** | 0.55 | 0.51 | 0.60 | 0.51 | 0.58 |
| **Right SLF** | 0.51 | 0.53 | 0.58 | 0.65 | 0.49 |
| **Left Uncinate** | 0.49 | 0.60 | 0.53 | 0.51 | 0.55 |
| **Right Uncinate** | 0.56 | 0.56 | 0.56 | 0.55 | 0.47 |
| **Left Arcuate** | 0.58 | 0.45 | 0.45 | 0.53 | 0.47 |
| **Right Arcuate** | 0.56 | 0.49 | 0.53 | 0.55 | 0.49 |

| **ACCURACY FEMALE PATIENTS ONLY** | | | | | |
| --- | --- | --- | --- | --- | --- |
| **TRACT** | **FA** | **MD** | **RD** | **AxD** | **ALL** |
| **Left Thalamic Radiation** | **0.78** | **0.56** | **0.66** | **0.69** | **0.69** |
| **Right Thalamic Radiation** | **0.63** | **0.66** | **0.69** | **0.56** | **0.59** |
| **Left Corticospinal** | **0.63** | **0.56** | **0.63** | **0.72** | **0.66** |
| **Right Corticospinal** | **0.59** | **0.63** | **0.56** | **0.63** | **0.59** |
| **Left Cingulum Cingulate** | **0.69** | **0.53** | **0.44** | **0.56** | **0.69** |
| **Right Cingulum Cingulate** | **0.59** | **0.56** | **0.59** | **0.69** | **0.53** |
| **Left Cingulum Hippocampus** | **0.69** | **0.63** | **0.66** | **0.63** | **0.72** |
| **Right Cingulum Hippocampus** | **0.66** | **0.75** | **0.66** | **0.69** | **0.69** |
| **Callosum Forceps Major** | 0.59 | **0.56** | **0.59** | **0.66** | **0.56** |
| **Callosum Forceps Minor** | **0.69** | **0.63** | **0.63** | **0.66** | **0.72** |
| **Left IFOF** | **0.66** | **0.59** | **0.66** | **0.59** | **0.63** |
| **Right IFOF** | **0.69** | **0.69** | **0.66** | **0.88** | **0.69** |
| **Left ILF** | **0.69** | **0.69** | **0.66** | **0.69** | 0.53 |
| **Right ILF** | **0.63** | **0.63** | **0.69** | **0.72** | **0.53** |
| **Left SLF** | **0.72** | **0.59** | 0.59 | **0.69** | **0.66** |
| **Right SLF** | **0.66** | **0.63** | 0.59 | **0.72** | **0.63** |
| **Left Uncinate** | **0.59** | **0.78** | **0.66** | **0.66** | **0.75** |
| **Right Uncinate** | **0.69** | **0.59** | **0.63** | **0.59** | **0.56** |
| **Left Arcuate** | **0.69** | **0.63** | **0.72** | **0.69** | **0.66** |
| **Right Arcuate** | **0.69** | **0.66** | **0.72** | **0.56** | **0.63** |

Values in bold indicate accuracies greater than obtained from classifiers with sexes combined (see Table 2 in full text)
